# Neural Circuits for Social Interactions: From Microcircuits to Input-Output Circuits

**DOI:** 10.3389/fncir.2021.768294

**Published:** 2021-10-29

**Authors:** Sen Xu, Ming Jiang, Xia Liu, Yahan Sun, Liang Yang, Qinghu Yang, Zhantao Bai

**Affiliations:** Shaanxi Engineering and Technological Research Center for Conversation and Utilization of Regional Biological Resources, College of Life Sciences and Research Center for Resource Peptide Drugs, Yanan University, Yanan, China

**Keywords:** social interaction, microcircuit, input-output circuit, medial prefrontal cortex, hippocampus, amygdala

## Abstract

Social behaviors entail responses to social information and requires the perception and integration of social cues through a complex cognition process that involves attention, memory, motivation, and emotion. Neurobiological and molecular mechanisms underlying social behavior are highly conserved across species, and inter- and intra-specific variability observed in social behavior can be explained to large extent by differential activity of a conserved neural network. However, neural microcircuits and precise networks involved in social behavior remain mysterious. In this review, we summarize the microcircuits and input-output circuits on the molecular, cellular, and network levels of different social interactions, such as social exploration, social hierarchy, social memory, and social preference. This review provides a broad view of how multiple microcircuits and input-output circuits converge on the medial prefrontal cortex, hippocampus, and amygdala to regulate complex social behaviors, as well as a potential novel view for better control over pathological development.

## Introduction

Social interaction refers to social activities in which individuals communicate with each other and conduct material and spiritual exchanges under certain conditions. It is an indispensable and complex behavior for many species, and it is essential for the survival and reproduction of animals (McGraw and Young, 2010). Although there have been many studies on the molecular and biological mechanisms related to social behavior, the exact neural circuit mechanisms are still unclear.

Based on brain imaging technology research, a greater understanding of anatomical structure of human brain has been garnished, especially the recent deepening of research on the social brain (Battle, 2013). The social brain is a general term for brain regions related to the process of understanding the social cognition of others and is mainly composed of the medial prefrontal cortex (mPFC) (Nie et al., 2018; Abe et al., 2019), hippocampus (HPC) (Moadab et al., 2017; Madiha and Haider, 2019; Rytova et al., 2019), amygdala (AMG) (Mihara et al., 2017; Stilling et al., 2018), anterior cingulate cortex (ACC) (Guo et al., 2019), inferior frontal gyrus (IFG) (Liu et al., 2015), superior temporal sulcus (STS) (Isik et al., 2017), and anterior insula (AI) (Bellucci et al., 2018). The social brain is the brain foundation of social behavior and plays a crucial role in social communication and mutual understanding. It involves the recognition of facial and body expressions, cognition of the thoughts and feelings of other people, prediction of next behavior and adjust communication with others, etc. (Frith and Frith, 2007; Blakemore, 2008). Social behavior is jointly regulated by multiple brain regions and neural circuits (Okuyama et al., 2014; Walum and Young, 2018). Studies have shown that the change of neuronal activity in mPFC (Nie et al., 2018; Abe et al., 2019), HPC (Moadab et al., 2017; Madiha and Haider, 2019; Rytova et al., 2019), AMG (Mihara et al., 2017; Stilling et al., 2018), ACC (Guo et al., 2019), IFG (Liu et al., 2015), cerebellum (Carta et al., 2019), STS (Isik et al., 2017), and AI (Bellucci et al., 2018) are closely related to expression of social behaviors, such as social fear, social emotion, social memory, and social preference. Therefore, exploring the precise mechanisms of the social brain and its neural circuits in social regulation is key to understanding the functional structure of the social brain.

This review will focus on the functions of the mPFC, HPC, and AMG regarding their role in social-related neural circuits. We will illustrate these neuronal networks at the levels of microcircuits, input circuits, and output circuits, assembling the accumulated knowledge to date to form a model of neuronal circuits regulating interpersonal interactions.

## Neural Microcircuits of Social Interaction

Within brain regions, the connective projections between excitatory neurons and inhibitory interneurons (INs) form neural microcircuits and participate in the regulation of different social behaviors, such as social fear, social hierarchy and dominance, social exploration, social memory, and social preference. Interneurons are heterogeneous in types and functions, which gives them the ability to fine-tune neural networks. Lim et al. reviewed over 50 different types of GABAergic neuron in the cerebral cortex. Parvalbumin-positive (PV), somatostatin-positive (SST), and vasoactive intestinal peptide-positive (VIP) GABAergic interneurons are the most widespread class of interneurons (Lim et al., 2018). Activation of VIP interneurons elicit inhibitory postsynaptic currents (IPSCs) from a large fraction of SST interneurons. While activation of VIP-elicited IPSCs in a smaller fraction of PV interneurons display stronger short-term depression, which receive strong inputs from principal cells and form inhibitory synapses with the soma, axon initial segment, and proximal dendrites of projection cells (Pi et al., 2013; Duvarci and Pare, 2014). Conversely, only a small fraction of pyramidal neurons responded to VIP activation, suggesting that pyramidal neurons are a minor monosynaptic target of the VIP population (Pi et al., 2013). Muller et al. (2007) reported that the high proportion of synaptic inputs from SOM terminals to the distal compartment of pyramidal cells in the BLA. In addition, following SST inactivation, the firing rate was largely increased in fast-spiking PV neurons upon approaching a social target. Simultaneously, the pyramidal cells showing increased spiking activities during the social approach were significantly reduced (Xu et al., 2019).

Abnormalities in GABAergic interneurons and inhibitory synaptic transmission in the social brain are associated with social behaviors. Bicks et al. (2020) reported that proper dorsomedial prefrontal cortex (dmPFC) PV activity is physiologically necessary for normal social behaviors. In addition, mPFC SST neurons were reported to suppress the activity of PV cells, inducing a disinhibition of local pyramidal cells that is crucial for aversive conditioning to a socially conditioned stimulus (Xu et al., 2019). Using chronic single-unit recording with optogenetic-tagging technique, Liu et al. (2020) shown that PV and SST neurons were significantly activated during real-time social interaction in mice. In addition, optogenetic activation of either PV or SST neurons at low gamma frequencies enhanced low gamma power and produced a prosocial effect (Liu et al., 2020). In a behavioral paradigm designed to test for preference toward social stimuli displaying altered affective states, SST neurons were reported to show such state-dependent specificity (Scheggia et al., 2020). Chemogenetic selective activation of PV-positive neurons have also been demonstrated to suppress neuronal activity in DG and greatly impair the social memory of mice (Zou et al., 2016). Restoring the excitability of inhibitory interneurons and reducing the excitability of granule cells in DG can improve the social exploration deficits in autistic-like mice (Kaplan et al., 2017). In addition, optogenetic excitation or inhibition of vCA1-PV neurons destroys social memory retrieval (Deng et al., 2019).

The excitatory and inhibitory neurons in the microcircuit jointly regulate social behaviors through input-output projections. Studies have indicated that a proper dose of the retinoic acid induced 1 (*RAI1*) gene is required in both excitatory and inhibitory neurons to control social interactions (Huang et al., 2018). Crossing *Rai1^*STOP*/+^* mice with *Vglut2^*Cre*^* or *Vgat*^*Cre*^ mice to restore Rai1 level in excitatory or inhibitory neurons alone was not sufficient to normalize social interaction. However, crossing *Rai1^*flox/flox*^* mice with *Vglut2^*Cre*^* or *Vgat*^*Cre*^ mice to delete one copy of *Rai1* expression in excitatory or inhibitory neurons often produced offspring, which presented as social losers in dominance-submission challenge tests. Recently, based on research in autism-like Shank3 heterozygous mice, which show global developmental delays, and which display behavioral and cognitive features resembling autism spectrum disorders (ASDs), it was observed that the amplitude of the miniature excitatory postsynaptic currents (mEPSC) of the pyramidal neurons in CA1 were reduced, and the input-output (I/O) relationship of CA1 synapses were also significantly reduced (Bozdagi et al., 2010). Specific inactivation of CA2 pyramidal neurons by injection of adeno-associated virus in Amigo2-Cre mice does not affect the exercise ability, anxiety-like behavior, spatial memory, episodic fear memory, or sound-dependent fear memory of the mice, but affects their social interaction behaviors and significantly reduces social preference (Hitti and Siegelbaum, 2014). In addition, ventral CA3 excitatory neurons mediate the coding of social memory (Chiang et al., 2018), whereas CA3 pyramidal neurons and inhibitory interneurons form feedforward and feedback microcircuits to regulate neuronal excitability, control the synchronization and oscillation of neurons, and participate in the coding of spatial memory and episodic memory (Rebola et al., 2017). Optogenetic activation of GABAergic neurons in the posterior dorsal subdivision of medial amygdala (MeApd) can trigger social grooming, sniffing, mounting, and attack behaviors, whereas photostimulation of glutamatergic neurons interrupt naturally-occurring attack behavior and suppress naturally-occurring mounting behavior (Hong et al., 2014). Recent work by Kuerbitz et al. (2018) showed that the zinc finger transcription factor *Tshz1* mouse mutants exhibit severely reduced numbers of intercalated cells (ITCs), which modulate the activity of BLA neurons. As a result, mice exhibit shorter latency to enter a partner’s portion of the cage, yet display impaired social interactions (Kuerbitz et al., 2018). Therefore, the balance between the activity of excitatory and inhibitory neurons in the brain controls the output of the neuronal circuits that regulate our behavior.

Changing the activation of glutamatergic and GABAergic neurons in the mPFC, HPC, and AMG affects different aspects of social behaviors, yet jointly participate in the regulation of social interaction. Precise analysis of the microcircuit construction between different types of neurons and the correlation with social interaction will help to clarify the social regulation of these nuclei and lay a foundation for neural microcircuit-based diagnosis and treatment for psychosocial disorders. In addition to the microcircuits in each brain area, the mutual projection relationship between different brain areas also constitutes different input and output circuits, which participate in the regulation of social behaviors. Therefore, exploring the neural network structure formed by the input and output loops of different brain regions is necessary for understanding the process of social regulation.

## Social Networks for Social Interactions

Social networks refer to social contacts among individuals, which are stable systems formed through social interactions. It is well established that mPFC, HPC, and AMG are all involved in the regulation of social interaction behavior, and they also form social networks with each other. Here, we focus on the social exploration, social memory and preference, social hierarchy, and dominance behaviors and summarize the input and output circuits under these behaviors.

### Neural Input-Output Circuits of Social Exploration

**FIGURE 1:**
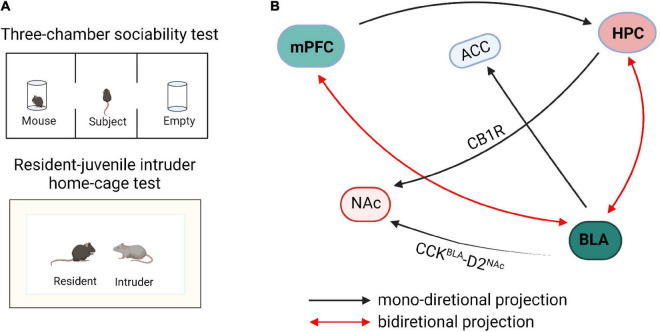
Schematic diagram of neural circuits within the social exploration. **(A)** The paradigm diagrams of social exposure behaviors. **(B)** The neural networks of social exploration. mPFC, medial prefrontal cortex; HPC, hippocampus; BLA, basolateral amygdala; NAc, nucleus accumbens; ACC, anterior cingulate cortex; CB1R, cannabinoid type 1 receptor; CCK, cholecystokinin.

During social interactions, animals must continuously collect and interpret sensory information related to the identity and sex of a partner, as well as its reproductive or dominance status and affective state (Chen and Hong, 2018): this behavior is called social exploration. Social exploration dictates the need for continuous monitoring of the social environment and dynamic modulation of behavior, as the response of the social partner will generate new sensory information and error signals to further guide the selection of optimal responses. The outcomes of repeated explorations can then drive long-term circuit plasticity that will influence subsequent behavioral responses in future social encounters (Insel and Fernald, 2004; Zhou et al., 2017). There are multiple neuronal projections formed between mPFC, HPC, and AMG (Figure 1), and it has been proven that they jointly or independently regulate social exploration behaviors. However, the precise construction of these networks and their regulation of social exploration behavior is still unclear and more research is needed.

GABAergic neurons and glutamatergic neurons in the input and output circuits of mPFC participate in social exploratory behavior. Li et al. (2018) reported that transitional immune activation before and after childbirth causes a significant decrease in social time, which is mainly due to the increased glutamatergic synaptic strength of mPFC projecting to the basolateral amygdala (BLA). Inhibitory synapses on PNs, which are formed by GABAergic interneurons, provide a strong feedforward inhibition in the mPFC-BLA pathway leading to an imbalance of excitatory/inhibitory microcircuits in the BLA. Kim et al. (2020) reported that optogenetic activation of mPFC-BLA disrupted socially induced neuronal activity and lead to abnormal social exploratory behaviors. In contrast, optogenetic inhibition of this circuit reversed the social behavior defects in mice (Kim et al., 2020). Conversely, optogenetic activation of BLA inputs to mPFC reduced social interaction and social exploration in the resident-intruder test, whereas inhibition facilitated social interaction and exploration behavior (Felix-Ortiz et al., 2016). These findings revealed that the BLA-mPFC circuit has a bidirectional regulatory effect on social exploration behaviors. Furthermore, the homeostasis of neurons and glial cells of the mPFC-HPC circuit coordinate and regulate social interaction. In an autism model induced by valproic acid (VPA), it has been observed that the spatial arrangement of neurons is disordered and the expression of astrocytes is increased in mPFC and hippocampus (Codagnone et al., 2015). Studies have shown that early hippocampal injury causes social disorders, development-specific increases in pro-inflammatory cytokines, and reduction in the expression of transforming growth factor beta 1 (TGF-β1). Administration of systemic recombinant TGF-β1 alleviates the social interaction and attenuates dendritic spine loss in mPFC layer 3 pyramidal neurons and suggests that mPFC TGF-β1 may alleviate social disorders caused by hippocampal injury (Joseph et al., 2018).

In the HPC-AMG circuit, the decreased GABA expression remarkably reduces the social exploratory time between caged mice and leads to anxiety-like behaviors (Bristow et al., 2020). The transmission of cannabinoid type 1 receptor (CB1R) from the hippocampus to nucleus accumbens (NAc) can significantly enhance the activity of NAc neurons, thereby blocking the transmission of AMPA/NMDA receptors and significantly reducing the social interaction index of mice (Loureiro et al., 2016). Optogenetic inhibition of BLA to ventral hippocampus (vHPC) projection urged mice to spend more time exploring the intruder in the resident-juvenile intruder home-cage test, whereas excitation of this projection caused mice to spend less time investigating the juvenile intruder in the resident-intruder procedure and also enhanced self-grooming behaviors (Felix-Ortiz and Tye, 2014). Further research showed that optical inhibition of the BLA-vCA1 circuit increased mouse social investigation time in the resident-invader paradigm, whereas optical activation of this circuit decreased the social investigation time (Felix-Ortiz et al., 2013). These results indicate that the BLA-vHPC circuit is closely related to social investigation behaviors and the transmission of glutamatergic signals is the key factor in social exploration.

Histologically, the majority of the neurons in the rodent BLA are glutamatergic pyramidal cells, which are predominantly projection neurons, whereas approximately 18% are GABA interneurons (Ferri et al., 2016). In addition, social exploratory deficits related to the functional connection between the amygdala and subgenual anterior cingulate cortex (sACC) are found in individuals with ASD who exhibit a negative relationship between amygdala-sACC connectivity and social dysfunction (Velasquez et al., 2017). More specifically, in individuals with ASD, less severe social dysfunction is related to more amygdala-sACC connectivity (Velasquez et al., 2017). Furthermore, the circuit of amygdala to NAc is also important in regulating social investigation behaviors (Wei et al., 2015; Varghese et al., 2017). Optogenetic activation of BLA-NAc glutamatergic circuit decreased mouse exploration time in the social chamber in a three-chamber test and mice spent less time in sniffing or investigating the cup in which the target mouse was contained (Folkes et al., 2020). Additionally, optogenetic inhibition of BLA-NAc circuit markedly increased social exploratory behavior in the Shank3B knockout mouse, an ASD model with substantial social interaction impairment (Folkes et al., 2020). Glutamatergic neurons which express cholecystokinin (CCK) in BLA negatively regulate the D2 type intermediate dendritic spine neurons in NAc (Shen et al., 2019). The excitatory transmission of CCK^BLA^-D2^NAc^ circuit is selectively enhanced in mice under social stress, and optical inhibition or activation of this circuit bidirectionally regulates susceptibility to social stress. These data reveal the importance of the excitatory neurotransmission of the BLA-NAc circuit in regulating social functions. Herein, BLA projections to the hippocampus, prefrontal cortex, NAC, and sACC are all involved in social behaviors, but whether these circuits are specific to social exploratory behavior is still unclear and needs further verification. The hippocampus regulates the mineralocorticoid receptors in amygdala through outward inhibitory signals, which drives social regulation in a sex-specific manner (Ter Horst et al., 2014). In addition, mice spend a greater amount of time actively avoiding their partner when the left hemisphere amygdala is kindled (Fournier et al., 2020). Optogenetic and electrophysiological studies revealed that contralateral BLA input results in synaptic facilitation of BLA neurons, thereby intensifying responses to cortical and thalamic stimulations. Pharmacological and chemogenetic inhibition of contralateral BLA connectivity effectively reduces investigation time in reciprocal social interactions (Huang et al., 2019), suggesting that BLA contralateral facilitation is required for social exploratory activities.

### Neural Input-Output Circuits of Social Memory and Preference

Social memory is the key to establishing relationships between individuals and mounting appropriate behaviors based on previous encounters. Social preference consists of the behavioral characteristics of distinguishing a novel conspecific from a familiar one, which is evolutionarily well conserved because of its importance for survival (Lu et al., 2018). Many brain regions, especially the HPC, mPFC, and AMG, have been reported to be related to social memory and the neural circuits formed between them regulate the formation, acquisition, storage, and retrieval of social memory.

The sub-circuits of mPFC activates in social memory and social preference behaviors. Huang et al. (2020) found that sustained closed-loop optogenetic activation of PL-BLA circuitry induces social impairment, corresponding to a negative emotional state as revealed by real-time place preference behavioral avoidance. When mice received light activation to the PL-BLA circuit, they lacked a social preference and spent equal time in the social and non-social chambers (Huang et al., 2020). Moreover, spatially specific manipulation of PL-NAc neurons bidirectionally regulated social-spatial learning, as assayed with a social conditioned place preference paradigm. Inhibition of PL-NAc neurons disrupted social-spatial learning, whereas activation of PL-NAc neurons generated an enhanced preference for the social zone associated with stimulation (Murugan et al., 2017).

**FIGURE 2:**
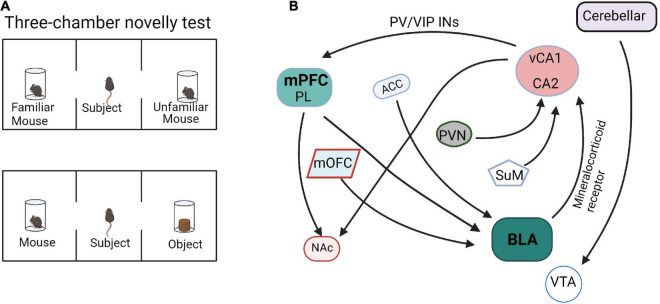
Functional connectivity within the social memory and preference circuits. **(A)** The paradigm diagrams of social memory and preference behaviors. **(B)** The neural circuits of social memory and preference. mPFC, medial prefrontal cortex; PL, prelimbic cortex; HPC, hippocampus; BLA, basolateral amygdala; mOFC, medial orbitofrontal cortex; NAc, nucleus accumbens; PVN, paraventricular nucleus; ACC, anterior cingulate cortex; SuM, supramammillary nucleus; VTA, ventral tegmental area.

Sun et al. (2020) showed that inhibition of vHPC or the direct projection from vHPC to mPFC caused mice to lose the distinction between familiar and novel mice, thus indicating that vHPC and vHPC-mPFC circuit mediates social memory. In addition, three major GABAergic neurons in mPFC, which are PV+, somatostatin positive (SST+), and vasoactive intestinal peptide positive (VIP+), all receive direct inputs from vHPC. Activation of PV + neurons in mPFC, but not mPFC SST + neurons or inhibiting VIP + neurons, can rescue the social memory impairment caused by vCA1 inhibition. It was suggested that the signal projected by vCA1 may affect the formation of social memory by overactivation of PV + neurons or overinhibition of VIP + neurons in the mPFC (Sun et al., 2020). The multiple input and output networks of mPFC mediate social memory behaviors (Figure 2), especially PL-BLA, PL-NAc, and vCA1-mPFC, but the refined network construction is still unclear and needs to be further explored.

The input and output circuits of the hippocampus play important roles in social memory (Figure 2). The dorsal CA2 (dCA2) has been shown to be necessary for the formation, encoding, consolidation, and recall of social memory (Donegan et al., 2020). Meira et al. (2018) reported that dCA2 participates in social memory by providing an excitatory input to the same vCA1 subregion that projects to the NAc shell. Moreover, the vHPC-NAc projections were essential for social discrimination behavior. Tonegawa and his college reported that optogenetic activation of the vCA1-NAc shell projections during social interaction with a novel mouse disrupted social discrimination and greatly reduced the sniffing duration (Okuyama et al., 2016). As mentioned above, the signal projected by vHPC can affect social memory by overactivation PV + neurons or inhibition VIP + neurons in mPFC (Sun et al., 2020). In addition, the hippocampus-amygdala projection is also involved in social memory regulation. Through behavioral experiments, it was found that selectively reducing the expression of mineralocorticoid receptor (MR) in the amygdala can significantly increase the interaction with unfamiliar mice, whereas activation of MR cause mice to lose the ability to recognize familiar or unfamiliar mice. Further, the hippocampus can regulate the expression of MR in the amygdala through outgoing inhibitory signals and participate in social memory regulation in a sex-specific manner (Ter Horst et al., 2014). Felix-Ortiz and Tye determined that optogenetic activation of BLA-vHPC projections reduced social behaviors as shown in the resident-juvenile intruder procedure. This was indicated by decreased time exploring the intruder and in the three-chamber sociability test by decreased time spent in the social zone (Felix-Ortiz and Tye, 2014). Additionally, studies have shown that targeted excitation of the hypothalamic paraventricular nucleus (PVN) projecting to CA2 vasopressin neurons during social memory acquisition, but not memory retrieval, dramatically prolonged social memory in mice from 30 min after a single encounter to at least 7 days (Smith et al., 2016). Another work reported that the hypothalamic supramammillary nucleus (SuM)-CA2 circuit is preferentially activated by novel social encounters (Chen et al., 2020).

Fascinatingly, the amygdala input circuits are also involved in social preference behavior. The neuronal activity of the medial orbitofrontal cortex (mOFC)-BLA projection increases prior to the initiation of social interaction as observed by the difference in neuronal activity presented 0.5 s before zone entry in a social interaction test. Inhibition of mOFC-BLA projection also reduced the interaction time in social novelty investigation behaviors (Li et al., 2021). These results indicated that the increased neuronal activity in mOFC-BLA projection may predict the initiation of a social interaction. In addition, the ACC-BLA circuit is also involved in the regulation of social behavior. In three-chamber tests, the mice with optogenetic inhibition of ACC inputs to the BLA did not show the expected avoidance behavior to the aggressive mouse and significantly improved the formation of social preferences (Allsop et al., 2018; Chang and Dal Monte, 2018). Although many BLA input and output networks are involved in the process of social behaviors (Figure 2), the specific neuronal and molecular architecture is still unclear.

Moreover, the cerebellum also involved in social preference. Optogenetically silencing the cerebellar-ventral tegmental area (VTA) projections continuously was as effective in preventing the expression of the social behavior in the three-chamber task as when the optical inhibition was applied only when the mouse was in the social chamber (Carta et al., 2019).

### Neural Input-Output Circuits of Social Hierarchy and Dominance

**FIGURE 3:**
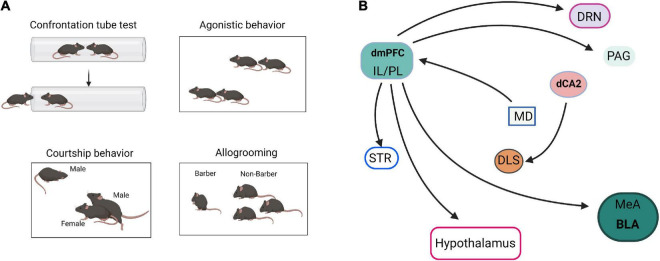
Neural pathways of social dominance. **(A)** The paradigm diagrams of social dominance behaviors. **(B)** The neural circuits of social dominance. dmPFC, dorsomedial prefrontal cortex; IL, infralimbic cortex; PL, prelimbic cortex; MeA, medial amygdala; BLA, basolateral amygdala; dCA2, dorsal CA2; DRN, dorsal raphe nucleus; MD, mediodorsal nuclei of thalamus; PAG, periaqueductal gray; STR, striatum; DLS, dorsal lateral septum.

Social hierarchy and dominance determine the right to access resources and profoundly affect survival, health, reproductive success, and various behaviors (Wang et al., 2011). Hu’s laboratory reported that mPFC is involved in establishing social hierarchy and that social rank is plastic, which can be tuned by changing the synaptic strength in mPFC pyramidal cells (Wang et al., 2011). Furthermore, Noonan et al. (2014) reported that individual social status in a hierarchical group was positively correlated with amygdala size. Hong et al. (2014) showed that stimulation of MeA GABAergic neurons elicits aggressive behavior, whereas stimulation of MeA glutamatergic neurons suppresses aggressive behavior. These results suggest that mPFC and AMG are the main control centers of social hierarchy and dominance (Figure 3).

In addition, social hierarchy and dominance are also regulated by multiple neural circuits. Hu’s laboratory reviewed the neural circuits that control social status (Wang et al., 2014; Zhou et al., 2018). The projection of mPFC to BLA, dorsal raphe nucleus (DRN), hypothalamus, striatum, and periaqueductal gray matter (PAG) are collectively involved in establishing social status (Wang et al., 2014). Selective optogenetic activation of the mediodorsal thalamus (MD)-dorsomedial prefrontal cortex (dmPFC) circuit is sufficient to induce tube test winning, highlighting the importance of this pathway in dominance behavior (Zhou et al., 2017). Dulka et al. (2018) reported that dominant hamsters selectively activate the IL-BLA neuronal projection compared with subordinates and naïve controls. However, dominant hamsters also activate a PL-BLA/CeA neural projection during social defeat stress significantly more than subordinates (Dulka et al., 2018). Leroy et al. (2018) reported that specific silencing of CA2 and CA2-dorsal lateral septum (DLS) projection by inhibitory G-protein coupled receptor hM4Di in Amigo2-Cre mice significantly decreased the fraction of mice that engaged in attack in the resident-intruder paradigm. These results indicated that dCA2 promotes aggression, at least in part, through its projections to the DLS.

## Concluding Remarks and Future Directions

With the advancement of technologies, such as neural tracing, optogenetics, and molecular imaging, social neural networks can be continuously analyzed at greater resolution. Regulation the abnormal input and output signals in these brain regions will become important therapeutic targets to effectively treat psychosocial disorders. Herein, we summarized the microcircuits and input-output network among mPFC, HPCs, and AMG, which are essential for social behaviors. We characterized the anatomical micro-connections between the mPFC, HPCs, and AMG, which are formed with PV, SST, VIP-GABAergic INs, and glutamatergic neurons. These microcircuits form feedforward and feedback circuit activations in response to social behaviors. We also analyzed the input and output loops of social exploration, social memory and preference, and social hierarchy and dominance based on mPFC, HPCs, and AMG. Dissecting the function of these microcircuits and input-output networks is valuable for understanding the mechanisms underlying multiple social behavioral impairments associated with neuropsychiatric diseases and may shed light on potential novel therapeutic targets for better interventions for psychopathologies. However, outstanding questions are raised, which must be addressed in future studies. Many circuits have overlapping functions, such as the ACC-BLA projection, which regulates memory and social interaction. Whether this crossover is regulated by specific neurons or molecules is unknown. Social behaviors are complex and changeable, and they are heterogeneous between different age groups and environments. There is still a long way to go to fine-tune our understanding of neuronal circuits and neuronal molecular mechanisms.

## Author Contributions

QY, SX, and ZB made substantial contributions to the conception and design of the review and gave final approval of the version to be published. YS, MJ, LY, XL, and ZB participated in writing the particular sections of the manuscript and approved the final version. QY and SX prepared the figures. All authors contributed to the article and approved the submitted version.

## Conflict of Interest

The authors declare that the research was conducted in the absence of any commercial or financial relationships that could be construed as a potential conflict of interest.

## Publisher’s Note

All claims expressed in this article are solely those of the authors and do not necessarily represent those of their affiliated organizations, or those of the publisher, the editors and the reviewers. Any product that may be evaluated in this article, or claim that may be made by its manufacturer, is not guaranteed or endorsed by the publisher.
